# Extracellular Vesicles as Biomarkers in Cardiovascular Disease; Chances and Risks

**DOI:** 10.3389/fcvm.2018.00113

**Published:** 2018-08-22

**Authors:** Annemiek Dickhout, Rory R. Koenen

**Affiliations:** ^1^Department of Biochemistry, Cardiovascular Research Institute Maastricht, Maastricht, Netherlands; ^2^Institute for Cardiovascular Prevention, Ludwig-Maximilians-Universität München, Munich, Germany

**Keywords:** microparticle, atherosclerosis, vascular remodeling, diagnostic, platelet, endothelial cell, monocyte

## Abstract

The field of extracellular vesicles (EV) is rapidly expanding, also within cardiovascular diseases. Besides their exciting roles in cell-to-cell communication, EV have the potential to serve as excellent biomarkers, since their counts, content, and origin might provide useful information about the pathophysiology of cardiovascular disorders. Various studies have already indicated associations of EV counts and content with cardiovascular diseases. However, EV research is complicated by several factors, most notably the small size of EV. In this review, the advantages and drawbacks of EV-related methods and applications as biomarkers are highlighted.

## Introduction

After having been disregarded for decades, extracellular vesicles (EV) are now in sharp focus as mediators of cell-to-cell communication and their importance is currently being investigated for many diseases. Progress is quick, particularly in the field of tumor biology, but also in other areas e.g., cardiovascular diseases, EV-related findings are gathering strong interest (1–3). Being identified as mere cellular “dust” in the 1960s, it has become clear that EV are much more than that. Extracellular vesicles are derived from parent cells and tissues and can be classified into roughly 3 classes: (I) microvesicles that originate from budding of the cell membrane, (II) exosomes, that have endosomal/intracellular organelle origin and (III) apoptotic bodies, that are generated during programmed cell death. However, there appears to be quite some overlap between those classifications, in a sense that microvesicles can be in the size range of exosomes or *vice versa* and/or carry supposed endosomal markers (4). All body fluids have been found to contain EV, hinting toward their abundance and their possible physiologic roles. In addition, given the specific cellular origin of the EV, they may contain interesting information, reflecting cellular functions or health status and thus ultimately revealing physiologic or pathophysiologic disease states. This would make EV excellent biomarkers. Whereas most biomolecular biomarkers (e.g., circulating proteins) do not contain information about the original cellular and tissue context, such information is often contained in EV, in the form of a palette of cell-specific surface markers and corresponding membrane-enclosed EV content. A sole determination of the cellular origin of EV e.g., in plasma could provide information about the nature, severity and prognosis of a particular disorder. A further perspective is offered by the analysis of the content of EV from patient specimen, since proteins or nucleic acids within EV can yield clues about the pathophysiologic mechanisms underlying the disease. The range of cardiovascular pathologies in which EV are suspected to play a role is very wide and still expanding. However, as the field of EV is still developing, the methods for optimal analysis of EV determination, purification, and analysis are heavily debated. As established biomarkers such as circulating proteins have validated methods (e.g., ELISA) and clearly defined pre-analytical variables (e.g., concerning sample preparation), these are much less clearly defined in the field of EV. In this overview, the current chances and risks of the use of EV as biomarkers will be discussed.

## Isolation and measurement of EV—are we getting closer to a gold standard?

### Isolation of EV

At first sight, the isolation of EV from biologic fluids appears rather straightforward. In theory, EV can be isolated to purity solely based on their physicochemical properties, because they are larger in size than the protein fraction yet smaller than whole cells, more dense than the lipid fraction, with a rather defined density range and quite robust due to their membrane encapsulation. In addition to these physical properties, EV possess a palette of surface markers specific for the parent cell type. Thus, there are many possible approaches for EV isolation (summarized in Table 1). The most widely used method remains centrifugation in its variants density gradient, differential and ultracentrifugation. The small size of EV is exploited in a number of sequential centrifugation steps, starting at low speed (300 g) to remove any intact cells and continuing at higher speeds to obtain fractions enriched in microvesicles (20,000 g) and exosomes (100,000 g). The sedimentation behavior of EV can be modified by using density gradients, allowing separation from proteins and other components. Although processing times can be quite long, a clear advantage of centrifugation is the possibility to process larger volumes, such as collected cell culture supernatants. The use of centrifugation is somewhat losing its popularity since the report of (lipo)protein and/or RNA-protein complex contaminations and loss of EV integrity after pelleting (5, 6).

**Table 1:** Overview of current isolation methods of EV from plasma.

**Isolation method**	**Principle**	**Advantages (A)/Drawbacks (D)**
Differential centrifugation	Sedimentation and/or density	A: Large volumes can be processed A: Widely used and facile method D: Risk of contaminations with plasma proteins D: Risk of EV aggregation/loss of integrity D: Quality depends on rotor type
Size exclusion chromatography	Size (largest elute first)	A: Well-accepted and facile method A: Good separation/recovery of EV A: Preserves integrity of EV D: Sample volumes small to medium D: Does not discriminate between EV origins D. results in sample dilution
Filters	Size	A: Processing of large volumes possible A: Allows size fractionation of EV D: Does not discriminate between EV origins D. risk of EV fragmentation
Microfluidics	Physical behavior of EV (size)	A: High sample recovery A: Suitable for small sample volumes A: Maintains EV integrity and properties D: Low sample throughput D: Not appropriate for large volumes D: Need of equipment for flow cell construction
ExoQuick^TM^	Precipitation using polyethylene glycol	A: Quick and straightforward sample handling A: Can be scaled up for larger samples A: Amenable to larger sample numbers D: Risk for contaminations with plasma proteins
Magnetic beads/affinity chromatography	Immuno-affinity by surface markers	A: Discriminates between EV origins A: Less contamination with plasma proteins A: Amenable to larger sample numbers D: Does not discriminate between EV sizes D: Often needs highly specific antibodies
Fluorescence-activated cell sorting (FACS)	Light scattering, fluorescence	A: Size- and surface marker-based sorting D: Long processing times D: Costly equipment

Gaining ground is size-exclusion chromatography (SEC), which is a quick and straightforward method for removing proteins and other contaminations from EV-containing fluids. Columns for SEC can be ordered commercially or easily cast in the lab, using common 10–50 mL syringes, SEC-medium and fine gauze to retain the gel in the column. Depending on the column length (and diameter), they can handle volumes in the mL range, although the use of a large column inherently leads to dilution of the EV-containing eluate. There is still some debate whether SEC-purified EV are free of (lipo)protein contaminations (7, 8) and it is recommended to perform appropriate controls. Conventional and ultrafiltration has also been used successfully to isolate EV (9). The principle is similar to SEC, as EV are separated based on their size properties. In a recent study, the use of a set of sequential filters with different pore sizes resulted in preparations of pure and size-defined EV (10). Although the filter system described in this study is custom made and thus not commercially available (as many published experimental setups), the approach appears to have potential to become a standard method for EV isolation.

Considering the increasing interest in microfluidics, it is not surprising that this principle is implemented in the design of novel EV isolation techniques [reviewed in Gholizadeh et al. (11)]. An elegant study made use of microfluidic mixing cells in combination with visco-elastic sheath fluids, in which the smaller exosomes were driven to the walls of the flow cells, while larger particles (microvesicles) and the sample fluid remained in the center of the flow path. The 3 fluid streams (from the 2 walls and the center) were collected separately and the 2 sample streams originating from the walls contained the isolated exosomes (12). The recovery of EV was found to be very high using this method, yet the actual setup is still designed for small sample volumes and low throughputs. Another widespread method for EV, and particularly exosome isolation is precipitation. Here, polymers (e.g., polyethylene glycol) and proprietary chemicals are used to cause specific precipitation of EV by disturbing the solvation layer around the membranes. Although this method is quickly and easily performed on low as well as higher sample volumes, there is a considerable risk of co-precipitation of contaminants.

All techniques mentioned above are based on the physicochemical characteristics of EV (e.g., size, surface potential, density) and inherently do not distinguish between different cellular origins. Yet since EV also share many cellular (surface) markers with their parent cells, the opportunity is created to specifically isolate EV using these markers (see below). Thus, the use of affinity chromatography or labeled magnetic beads is an attractive alternative or a complement to physical techniques such as centrifugation and SEC. One study took advantage of the binding affinity of EV for heparin and could enrich EV from biologic samples using heparin coupled to agarose, which is a commonly used reagent in protein purifications (13). Although this method is straightforward and resulted in highly enriched EV preparations, there are many abundant plasma and serum proteins with high affinity for sulfated glycosaminoglycans, e.g., antithrombin, CXCL4, and apolipoproteins. A higher level of specificity can be achieved by using antibodies against specific surface markers to isolate EV. Needless to say, this requires markers unique for a particular EV subset and corresponding antibodies with high specificity. The potential to separate exosomes from extracellular vesicles depends on the cellular origin of the EV preparations (e.g., the exosome marker CD9 is present both on exosomes and microvesicles from platelets). However, physicochemical separation principles can be used to enrich or deplete the EV preparations in/from exosomes prior to implementing an affinity-based isolation method. Alternatively, EV have been sorted using sorting by FACS, combining scattering and marker-based detection methods (14). However, apart from the limitations described below, a modern FACS sorter might not be accessible due to high costs and the processing times per sample might be quite long.

Taken together, there is as yet no golden standard for EV isolation. The optimal method of EV purification depends, as often, on the characteristics of the starting material and the demands of the downstream applications. The good news in this respect is that many, often multidisciplinary research groups are in the course of developing innovative methods to achieve high quality EV preparations.

### Measurement and characterization of EV

A similar story can be told for the measurement and isolation of EV. The distinct properties of EV can likewise be used to measure and characterize EV. A summary of the current methods is summarized in Table 2. In general, their small dimensions and rather heterogeneous size distribution rather hampers accurate measurement of EV (15). This particularly applies for flow cytometry, as older generation machines (that are still commonly used) generally lack the capability to (accurately) measure particles sized below 300 nm. Another complication is the low refractive index of EV and thus the rather low capability to scatter light in aqueous solutions. In addition, common cytometer optics poorly distinguish single EV from EV swarms, which complicates the exact determination of EV counts in samples (16). On the other hand, modern flow cytometers have optics that allow the measurement of single particles as small as 100 nm and combined with careful apparatus setup and parameter adjustment (the authors refer to www.exometry.com), reliable characterization of EV using flow cytometry is possible. This, combined with the use of fluorescently-labeled antibodies and the high throughput of the method, makes flow cytometry still a method of choice for EV determinations.

**Table 2:** Overview of current measurement principles of EV.

**Detection method**	**Principle**	**Advantages (A)/Drawbacks (D)**
Flow cytometry	Light scattering, fluorescence	A: Fast recording and high throughput A: Combined size and surface marker analysis D: Limited possibilities using older machines D: Artifacts possible due to swarm detection
Tunable resistive pulse sensing	Coulter effect (electrical resistance changes)	A: Feasible and accurate size determination D: Membranes may clog D: Accurate measurements are slow
Nanoparticle tracking analysis	Light scattering, (fluorescence)	A: Feasible and accurate size determination D: Requires careful calibration D: Accurate measurements are slow
Dynamic light scattering and Raman spectroscopy	(In-)elastic light scattering	A: Feasible and accurate size determination A: Raman yields information about composition D: Low throughput D: Requires high technical proficiency
Transmission electron microscopy	Transmission of accelerated electrons	A: Size and structure determination A: Yields impressive images D: Low throughput D: Requires high technical proficiency
Atomic force microscopy	Power exerted to cantilever	A: Size and structure determination A: Gives information about surface markers D: Low throughput D: Requires high technical proficiency
Enzyme-linked immunosorbent assay	Antigen binding to antibodies, fluori-/colorimetric	A: Highly specific and facile method A: High throughput D: Limited information about counts and size
Western blotting	Antigen binding to antibodies, chemiluminescence	A: Facile method for composition analysis A: Medium throughput D: Limited information about counts and size
Surface plasmon resonance	Proteins binding to ligands,	A: Highly specific and facile method A: High throughput D: Limited information about counts and size D: Rather expensive and specialized equipment

Two other commonly used techniques are tunable resistive pulse sensing (TRPS) and nanoparticle tracking analysis (NTA). The former is based on the current of ions over a permeable membrane with pores that have tunable size. Particles that block the pores result in increased membrane resistance, from which a distribution of size and numbers can be derived. The method is quite reliable over a range of EV sizes and concentrations, yet care should be taken not to clog the pores of the membrane or to apply excessive pressure (15). The detection of EV by NTA is based on light scattering combined with recording of their Brownian motion paths using a microscope-camera setup. Knowing the viscosity and temperature of the sample medium, the EV size can be derived from their diffusion coefficients calculated using the Stokes-Einstein equation. A prerequisite is that viscosity and temperature are exactly defined and that the machine is calibrated using particles that have similar size and scatter characteristics as the EV analyzed (15, 17). Nevertheless, the inaccuracies in EV counts obtained by NTA can be quite high, but protocols have been developed to minimize sample-to-sample variations (18, 19). A similar technique is dynamic light scattering, which like NTA is based on elastic Rayleigh scattering and makes use of the Stokes-Einstein equation for calculating the diffusion coefficients. A further with potential method is Raman spectroscopy, which is based on inelastic light scattering making this method attractive also for analyzing EV, since inelastic light scattering contains information about the molecular composition of the EV (17, 20). Drawbacks are the high technical and mathematical complexity of the method and the long processing times of the analyses.

The same may apply for electron microscopy. Although electron micrographs of EV preparations are recommended to be included in EV-focused publications, the proper sample preparation and recording of (transmission) electron micrographs of EV requires a high level of technical proficiency. The images do reveal useful information about EV ultrastructures and if combined with immunologic detection methods (immunogold labeling), even information about molecular composition may be obtained. An interesting evolution of electron microscopy is cryo-electron tomography, in which a series of electron micrographs is recorded at various angles and then composed to a 3D image (21). A recent study has proven cryo-electron tomography to be feasible for EV, at least those derived from platelets, revealing a surface covered with platelet receptors linked to the actin cytoskeleton (22). A final non-optical microscopy method worth discussing is atomic force microscopy [reviewed in Sharma et al. (23)]. The technique works by a microscopic tip at the end of a cantilever that scans (surface immobilized) biologic specimen, somewhat like a microscopic turntable. The force exerted on the tip and cantilever is used to reconstruct an image of the specimen. The microscope can be set in various modes, each with their specific properties for obtaining information. Major advantages of this technique are that it is optimally suited for nanoscale specimen and that both structural features and information about surface molecules can be recorded, as the tips can be functionalized with specific antibodies.

Besides physical (optical) techniques, EV can also be determined using biochemical (immunologic and enzymatic) techniques. By making use of the presentation of negatively charged phospholipids (notably phosphatidyl serine) on their surface, EV can be captured e.g., using immobilized annexin A5 and subsequent detection using the prothrombinase reaction, which is highly dependent on negatively charged phospholipids (24). However, since not all EV express phosphatidyl serine, a significant fraction might be missed using this method. In addition to enzymatic assays, immunologic methods or “bulk immunologic assays” (BIA) might also be applied (25). The many surface molecules presented on EV and their small size can be exploited to design specific ELISAs for EV subgroups. In addition, EV content can be resolved on SDS PAGE gels followed by western blotting. A more sophisticated method is surface plasmon resonance spectroscopy, which measures refractory index changes as a result of mass bound to a golden surface under flow conditions. When the surfaces are functionalized with specific antibodies against EV surface markers, EV can be measured specifically within biologic samples, as was recently demonstrated for endothelial EV (26). Although the above assays have the advantage that they allow the qualitative and specific analysis of EV, the results of BIA may be difficult, if not impossible, to translate to absolute counts and to particle sizes.

Similar to their isolation, the measurement of EV is accompanied by restrictions that lie in their small size and their physicochemical properties. Also here, a gold standard of EV determination is not clearly defined. Chances are offered by the exploitation of the palette of surface markers carried by EV combined with a technique that specifically scans particles in the correct size range. As technology stands now, flow cytometry still has the highest potential to become a standard method.

## Differentiation of the cellular origin of EV

As mentioned in the introduction, EV can originate from virtually every cell or tissue. In the vasculature, the primary cell types that would release EV eligible as biomarkers are red blood cells, platelets, leukocytes, and vascular cells. Among those cell types, red cells and platelets greatly outnumber the others. Thus, EV derived from red cells and platelets are common in plasma. Some studies provide an estimation of the relative numbers of EV from different cell types in blood, yet due to the uncertainties and variations (as outlined above), the authors refrain from listing numbers in this overview. For example, it is still debated whether the platelet-derived EV found in plasma actually originate from platelets or from megakaryocytes (27).

Crucial for a meaningful exploitation of circulating EV as biomarkers is the differentiation of cellular origin. Many studies use surface molecules that function as indicators of the parental cells of the EV investigated. Although many cells are successfully being typed using surface markers, the use of such markers might be accompanied by difficulties. First, EV and in particular exosomes, are much smaller, making their measurement by e.g., flow cytometry technically more challenging (see below). Second, while aggregates of whole cells can readily be excluded from a flow cytometric analysis, the exclusion of EV aggregates is hampered by the large variation of EV size, meaning that a pair of 2 smaller EV can have a size similar to another larger EV. This might give rise to seemingly double-positive EV, which are in fact aggregates composed of EV from different cellular origins. Third, cellular activation is often required for EV formation, yet is also accompanied by activation of proteases (e.g., of the ADAM family), as is reported for platelets and endothelial cells (EC) (28, 29). Residual protease activity on EV might lead to loss of surface markers in time, which is relevant as a physiologic factor, but also as a pre-analytical variable to be taken into account. The latter even impacts characterization of EV from isolated cells, as surface molecules may be lost during storage, giving rise to day-to-day variations. A fourth difficulty is the availability of cell-specific markers (and corresponding specific antibodies). Platelets and EC for example, share quite a number of surface markers (e.g., P-selectin, CD31, β_3_ integrins, thrombospondin, von Willebrand factor, and many more), which complicates a clear differentiation, particularly in samples where platelet-derived and EC-derived EV outnumber those from other sources. Platelet- and EC-derived EV are often distinguished by the use of a common (e.g., CD31) and a platelet-specific marker (CD41 or CD42), bearing the risk that platelet-derived EV with poor antibody binding (e.g., by loss of a marker) can falsely be counted as EC-derived EV. This also applies in cases that cell-specific EV need to be isolated from biologic fluids. Since the common techniques of differential centrifugation or size exclusion chromatography do not distinguish between EV from different cell types, the correct use of markers for the enrichment of cell-specific EV from platelet- or erythrocyte-derived contaminants or protein aggregates is crucial.

## Potential of EV as biomarkers—recent examples

Despite the above reservations, surface markers are widely used in studies exploring levels of particular EV in health and disease. An accurately adjusted flow cytometer with size calibration and optics suitable for small particle analysis considerably facilitates EV determination. The number of flow cytometers that are capable of measuring EV available on the market is increasing. By combining small particle measurement with specific surface markers, information about EV content in biologic fluids can be obtained with a satisfactory level of accuracy. Several reported markers used for the determination of EV from particular cell types are summarized in Table 3. Although a large variety of techniques for EV analysis is available (Table 2), almost every patient sample-based study has been performed using flow cytometry. A recent multicenter collaborative workshop was organized by the International Society on Thrombosis and Haemostasis (ISTH) Vascular Biology Standardization Subcommittee to evaluate a new, universal standardization protocol to measure platelet EV counts using flow cytometry (38). This new standardization protocol was based on side scatter (SSC) and forward scatter (FSC) of pre-defined beads rather than only FSC before, dependent on which parameter is used as the best resolving size parameter in specific flow cytometers present in the laboratories. The study showed that this bead-based assay has potential for standardization of measurement of platelet EV numbers, however this procedure is not suitable to measure particle size.

**Table 3:** Examples of markers used for the determination of the cellular origin of EV.

**Cell type**	**Markers**	**References**
Monocyte	AnxA5, CD11b, CD14, CD31, CD64, CD142	(30–32)
Lymphocyte	CD3, CD45	(31)
Neutrophil	AnxA5, CD35, CD66b, MPO	(32–34)
Platelet	AnxA5, CD31, CD41, CD42, CD61	(31)
Megakaryocyte	CD62P-, LAMP-1, full-length filamin A	(27)
Endothelial cell	VCAM-1, CD62E, CD144, CD31, CD41-, CD42-	(32, 35)
Red blood cell	AnxA5, CD235a	(36, 37)

*AnxA5, annexin A5; MPO, myeloperoxidase; LAMP, lysosome-associated membrane protein; VCAM, vascular cell adhesion molecule*.

Altered levels of EV were found in cohorts of patients with variety of cardiovascular diseases. Several recent reviews provide an excellent and comprehensive overview of the relevant studies (3, 7, 39–41) and highlights of original work are listed in Table 4. In general, the origin of the EV investigated in most studies is derived from 3 main cell types: endothelial cells, leukocytes, and platelets. This is not surprising, since all 3 cell types are in direct contact with the blood. In addition, the endothelial lining of the vessel wall constitutes a huge surface (approx. 7,000 m^2^) and in the case of (systemic) inflammation, cytokines may increase the activation state of the endothelium, giving rise to the release of numerous EV. The same counts for platelets, since their sheer numbers combined with their capability to release EV upon activation can result in steep increases in EV numbers during pathologic conditions such as arterial and venous thrombosis. When surface markers become more defined and the analysis techniques more refined, also EV from rare cell types that are underrepresented in biologic samples may be detected.

**Table 4:** Cardiovascular disorders (CVD) with involvement of EV.

**Pathologic setting**	**EV from cell types involved**	**References**
EV as risk factor for CVD	Endothelial cells	(14, 36, 42–48)
	Platelets	(14, 36, 42, 46–48)
	Leukocytes (unspecified)	(42, 46, 47)
	Monocytes	(46, 48)
	Lymphocytes	(43)
	Hematopoietic cells	(43)
	Smooth muscle cells	(43)
	Erythrocytes	(36)
Vascular calcification	Smooth muscle cells	(49–52)
	Macrophages	(53)
	Endothelial cells	(54)
	Platelets	(54)
	Leukocytes (unspecified)	(54)
Coronary artery disease and acute coronary syndrome	Endothelial cells	(37, 44, 55–58)
	Platelets	(44, 55, 57, 58)
	Erythrocytes	(37)
	Leukocytes (unspecified)	(55, 58)
	Monocytes	(31)

Most of the studies show a positive correlation between EV counts and the cardiovascular disorder investigated, regardless of the cell type of origin. This may reflect the common observation that cells show an increase release of EV after activation. EVs have shown to be upregulated in patients with endothelial dysfunction or atherosclerosis (42, 59), in patients with deep vein thrombosis or pulmonary embolism (60–62), in patients with cerebrovascular diseases(63–66) or in patients that show cardiovascular risk factors like type-2 diabetes mellitus (67), severe hypertension (68) or obesity (69). Determining the EV's parent cells harbors the possibility to obtain additional information about the pathophysiology of a particular disorder. For example, in a small case-control study of one of the study arms of the PREDIMED trial with participants following a Mediterranean Diet, the EV levels of different cell types were measured. Participants suffering a cardiovascular event (CVE) within 1 year of intervention, showed increased EV release from lymphocytes and smooth muscle cells. Participants that did not have a future CVE within the follow-up time showed reduced EV release from these cells (43). In a case-control study at the NIH Stroke program, even different subtypes of EVs from endothelial parent cells could be distinguished. This study compared endothelial cell-derived EV levels in 20 patients with a mild stroke (NIHSS score < 5) to the EV levels of 21 patients with moderate to severe stroke (NIHSS score ≥ 5), and to the levels of 23 age-matched healthy volunteers. Using flow cytometry, they observed significantly higher phosphatidyl serine^+^ EV counts in patients compared to the controls, and all endothelial derived EV counts were elevated in the moderate to severe stroke group compared to controls. In patients with acute ischemic stroke, three endothelial cell microparticle (EMP) phenotypes (Endoglin^+^ EMP, phosphatidyl serine^+^ EMP, and ICAM-1^+^ EMP) correlated significantly with brain lesion volume, with ICAM-1^+^ EMP (*P* = 0.002) showing the strongest correlation. These data combined suggest a possible role of endothelial-derived EV numbers as a biomarker for severity and brain lesion size in patients with ischemic stroke (64).

Endothelial dysfunction is an independent predictor of vascular disease. Therefore, quantitative measurements of CD31^+^/Annexin A5^+^ EVs were assessed by Sinning and colleagues in patients with stable coronary artery disease (CAD). EV levels were higher in patients that later developed a major adverse cardiovascular and cerebral event (44). This study also finds that the presence of diabetes and male gender are significantly positively correlated to the number of EVs, a factor that has to be taken into account during risk stratification.

In addition, since EVs can be purified, their numbers and contents can be enriched manifold, which opens the possibility for the identification and determination of biomarkers that were previously too dilute to be measured in biologic fluids. Some studies have exploited this to identify miRNAs with prognostic value for cardiovascular diseases (14, 36). EVs of thrombin-stimulated platelets have elevated levels of miR-223 in complex with Argonaute 2, are taken up by HUVEC cells *in vitro*, and regulate gene expression levels through regulatory elements in the 3′UTR region of two specific mRNAs (70). This is only one of the examples in which platelets can alter specific gene regulation in HUVEC cells.

Moreover, the proteins cystatin C, serpin G1 and F2, and CD14 found in EVs have been identified as potential biomarkers by Kanhai et al. in 2013, using the Athero-Express discovery cohort (71). This was the first large, single-center cohort of 1,060 patients, to describe the protein content of EVs is related to increased risk of secondary cardiovascular events. Increased levels of cystatin C, serpin F2, and CD14 were correlated to an increased risk of myocardial infarction, vascular events and all-cause mortality, whereas increased levels of CD14 was also correlated to an increased risk of the occurrence of an ischemic stroke. This study only takes total EV protein levels in account, and not the number of EV.

Another prospective single-center cohort study showed that the EV protein levels polygenic immunoglobulin receptor, cystatin C and complement C5a were independently associated with acute coronary syndrome (72). This study also indicates an important discrepancy between male and female patients, where male patients show a strong correlation between the aforementioned proteins and ACS, whereas female patients did not.

Patients at risk for CVD with high LDL levels are often treated with statins. Statins prevent cardiovascular events, possibly not only by reducing plasma LDL levels. The METEOR trial aims to determine the effect of rosuvastatin on subclinical atherosclerosis. Patient serum samples and LDL-EVs were analyzed for their protein content of von Willebrand factor (vWF), Serpin C1, and plasminogen. Rosuvastatin-treated patients have higher levels of plasminogen and vWF in LDL-associated EVs, serum plasminogen levels were also increased but to a lesser extent, and serum vWF levels were not increased (73). This study concludes that this could be a possible new intermediate between statin therapy and coagulation.

Possibly the most studied potential biomarker is the coagulation potential of EVs exposing tissue factor (TF^+^ EV) in cancer patients, to evaluate the relative risk on developing venous thromboembolism (VTE) which is a complication in many cancer patients. In a multinational, prospective cohort study, the procoagulant activity of TF^+^ EV was evaluated using an in-house TF^+^ EV activity assay based on fibrin generation. The TF^+^ EV activity was measured in patients with various types of advanced cancer, and correlated with the development of venous thromboembolism (VTE). A high fibrin generation test outcome was associated with a two-fold increased risk for VTE, with the strongest association in patients with pancreatic cancer (four-fold increase) compared to patients with other tumor types (1.5 fold increase). The activity of TF^+^ EV measured using the fibrin generation test correlated poorly with the more commonly used TF-dependent Xa assay (74). However, there are also studies that do not find correlation of EVs with risk for VTE (75), or only a correlation with mortality but not with thrombosis (76). Therefore the role of TF^+^ EV as biomarker for VTE in cancer patients remains a matter of debate. Not only EVs derived from cancer cells but also EVs from monocytes expose TF. Although platelets were found to express TF (77), EVs from platelets and erythrocytes lack TF but did induce thrombin generation in a FXII-dependent matter (78).

Despite these examples of protein content in or on EVs, most of the current studies are limited to absolute counts of EV from particular cell types. The number of studies showing correlations or associations of EV numbers with disease prognosis, severity or occurrence is steadily increasing. It must be noted that the majority of studies have a relatively low number of subjects included in the investigation. The largest study to date on EV numbers in CVD is the study of Amabile et al. (2014) where 844 individuals in the Framingham Offspring cohort were studied (45). In this cohort, endothelial-derived EVs were associated with the presence of several cardiometabolic risk factors, including higher triglyceride levels, hypertension, and metabolic syndrome. The highest correlation was found with elevated triglycerides. However, this study only focused on large vesicles ≥ 500 nm, thereby risking that a substantial amount of the sample consists of apoptotic bodies.

Another complication is the lack of standardization of sample processing and measurement, making it difficult to compare studies from different laboratories. The same counts for the selection of surface markers analyzed and the corresponding allocation to the EV's parent cell types. A final issue is the heterogeneity of the sample populations investigated, meaning that there are quite some indications that EV are associated with a particular cardiovascular disease, yet hard evidence for such association in large cohorts of defined subjects is still largely lacking. It has to be noted that the above complications largely apply for biomarker research in general.

## Concluding remarks

Without doubt, the potential of EV as biomarkers is considerable. They may contain information about the original tissues, the pathophysiologic context and the severity of disease. On the other hand, the field is still relatively young and the progress in technologic development for accurate analysis is somewhat lagging behind the desires and ambitions of the investigators working in the area. Still, there is increasing consensus about the standardization of sample preparation and analysis of EV, meaning that studies are becoming more and more reliable and comparison between study locations becomes increasingly feasible. Taken together, interesting and exciting times are awaiting us, as EV do seem to be a big step toward the highly anticipated liquid biopsies.

## Author contributions

All authors listed have made a substantial, direct and intellectual contribution to the work, and approved it for publication.

### Conflict of interest statement

The authors declare that the research was conducted in the absence of any commercial or financial relationships that could be construed as a potential conflict of interest.

The reviewer JT and handling editor declared their shared affiliation at the time of the review.
